# Cohort Profile Update: the Rotterdam Periconceptional Cohort—biomarkers, lifestyle intervention, and artificial intelligence

**DOI:** 10.1093/ije/dyag156

**Published:** 2026-08-19

**Authors:** Wietske A P Bastiaansen, Bruno M de Brito Robalo, Melek Rousian, Sam Schoenmakers, Sten Willemsen, Anton Koning, Esther Baart, Annemarie Mulders, Eric Steegers, Régine P M Steegers-Theunissen

**Affiliations:** Department of Obstetrics and Gynaecology, Erasmus MC, University Medical Center, Rotterdam, The Netherlands; Biomedical Imaging Group Rotterdam, Department of Radiology and Nuclear Medicine, Erasmus MC, University Medical Center, Rotterdam, The Netherlands; Department of Obstetrics and Gynaecology, Erasmus MC, University Medical Center, Rotterdam, The Netherlands; Department of Obstetrics and Gynaecology, Erasmus MC, University Medical Center, Rotterdam, The Netherlands; Department of Obstetrics and Gynaecology, Erasmus MC, University Medical Center, Rotterdam, The Netherlands; Department of Obstetrics and Gynaecology, Erasmus MC, University Medical Center, Rotterdam, The Netherlands; Department of Biostatistics, Erasmus MC, University Medical Center, Rotterdam, The Netherlands; Department of Pathology, Erasmus MC, University Medical Center, Rotterdam, The Netherlands; Department of Obstetrics and Gynaecology, Erasmus MC, University Medical Center, Rotterdam, The Netherlands; Department of Obstetrics and Gynaecology, Erasmus MC, University Medical Center, Rotterdam, The Netherlands; Department of Obstetrics and Gynaecology, Erasmus MC, University Medical Center, Rotterdam, The Netherlands; Department of Obstetrics and Gynaecology, Erasmus MC, University Medical Center, Rotterdam, The Netherlands

**Keywords:** pregnancy, predictors, three-dimensional imaging, virtual reality, first trimester, pregnancy, nutrition, homocysteine, digital lifestyle intervention, artificial intelligence

Key Features

- The ongoing Rotterdam Periconceptional Cohort (Predict study) is an open cohort, initiated in 2009 to investigate maternal and paternal periconceptional health and their impact on reproduction, pregnancy, and neonatal outcomes. In the pilot phase (2009–10), 233 pregnancies were included. In the study phase (2010–23), 3566 pregnancies (participants aged between 19 and 51 years) and 2891 partners were included.
- New is the analysis of periconceptional maternal and paternal biomarkers in subcohorts: renin–angiotensin–aldosterone system (RAAS) components, tryptophan metabolites, homocysteine, C-reactive protein (CRP), hair cortisol/cortisone, cell-free DNA methylation for placental epigenetic profiling, and morphokinetic parameters.
- The BEYOND subcohort was embedded to investigate associations between bariatric surgery and maternal, embryonic, fetal, and placental outcomes; and the PROMOTE subcohort explored maternal microbiota, immune responses, and one-carbon metabolism in association with obesity.
- First-trimester imaging biomarkers of the uteroplacental vascular skeleton were developed using computer-assisted image processing to assess placental development. Advanced biomarkers were automated using artificial intelligence (AI), with innovations in the four-dimensional Human Embryonic Brain Atlas for spatiotemporal brain analysis.
- Users of www.SmarterPregnancy.co.uk were included as a subcohort to assess the effectiveness of personalized digital lifestyle coaching on features of maternal health and embryonic development.

## The original cohort

Extensive research of the Developmental Origins of Health and Disease (DOHaD) has demonstrated associations between early-life conditions, perinatal outcomes, and long-term health and disease [1]. In the last decades, we have shifted the exposure window to the periconceptional period (14 weeks before to up and until 10 weeks after conception) covering key processes of gametogenesis, embryogenesis, and placentation [2]. Since then, parental health and lifestyle during this window have been associated increasingly with pregnancy complications and long-term cardiometabolic and developmental outcomes in offspring [2].

The Rotterdam Periconceptional Cohort (Predict study) was initiated to bridge the gap of other birth cohorts starting data collection in late pregnancy or at birth, neglecting the critical periconceptional period [3]. This hospital-based open cohort is embedded in tertiary care, with couples uniquely recruited in the periconception period. The cohort includes serial consultations and ultrasound scans using advanced virtual reality (VR) and computer-assisted image processing for assessment of embryonic, fetal, and placental growth and morphology. The cohort focuses on three research areas: (i) periconceptional parental health; (ii) reproductive performance and outcomes; and (iii) molecular mechanisms, especially one-carbon metabolism, epigenetics, cardiovascular, and inflammatory pathways. Our ultimate aim is to translate these scientific findings into preventive strategies and better care.

**Figure 1:**
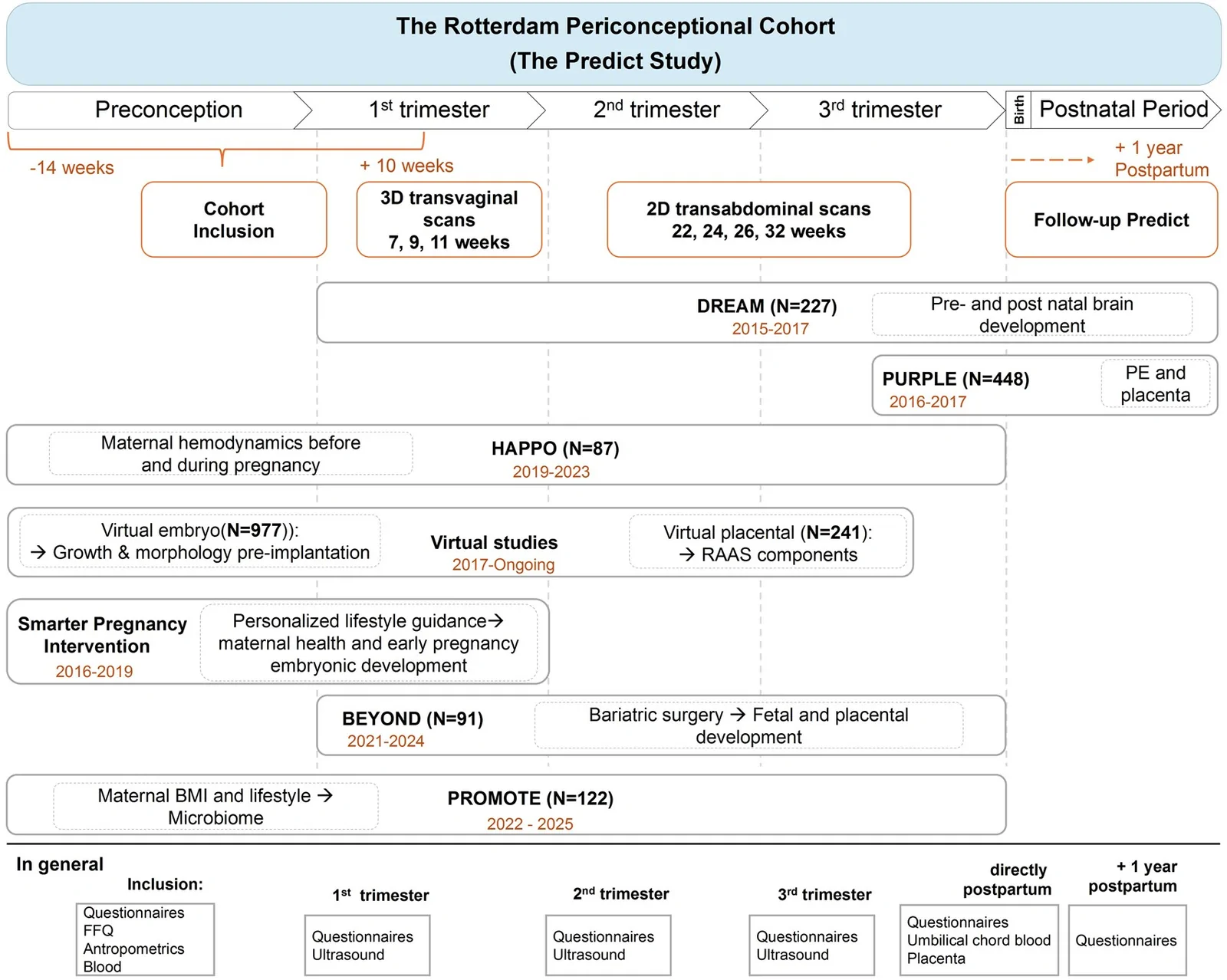
Cohort overview. Overview of the cohort design, including the timeline and embedded subcohorts. General assessments were conducted during the periconceptional period and pregnancy, as well as directly postpartum and at 1-year follow-up. Serial first-trimester two-dimensional (2D) and three-dimensional (3D) transvaginal scans were obtained at 7, 9, and 11 weeks. The DREAM subcohort (2014–15, *N* = 227) examined embryonic and fetal early brain development [4]. The Purple subcohort (2016–17, *N* = 448) focused on preeclampsia and placental complications [5]. The Virtual Placenta subcohort (2017–19, *N* = 241) investigated uteroplacental vascular development and function [6]. The HAPPO (Adaptation to Pregnancy and Placenta-related Outcome) subcohort (2019–23, *N* = 87) evaluated maternal haemodynamic adaptation to pregnancy [7]. The ongoing Virtual Embryoscope study (2017–present, *N* = 977 until August 2023) investigates pre-implantation embryo development [8]. The DREAM and Virtual Placenta subcohorts introduced additional 2D and 3D transabdominal imaging at 22, 24, 26, and 32 weeks. FFQ: food frequency questionnaire; PE: preeclampsia.

The cohort design, recruitment procedures, and original measurement protocol are described by Steegers-Theunissen *et al*. [3]. This cohort also embeds several subcohorts to address specific topics and research questions, as illustrated in Fig. 1.

## What is the reason for the new focus?

Since the last update, we expanded the inclusions to around 3800 pregnancies with data from both partners across the periconceptional and pregnancy periods [9]. In response to identified knowledge gaps in obesity, bariatric surgery, cardiometabolic adaptation, mental stress, and placental pathology, we broadened the cohort’s focus toward deeper mechanistic biomarker research by embedding several subcohorts.

In parallel, advances in artificial intelligence (AI) for medical image analysis created new opportunities to leverage our uniquely longitudinal first-trimester imaging dataset for the automated extraction of embryonic and placental imaging biomarkers.

To translate knowledge into practice, we investigated the clinical effectiveness of our lifestyle coaching program in a subcohort (www.smarterpregnancy.co.uk).

## What will be new areas of research?

### Biomarkers

The impact of preconceptional bariatric surgery on embryonic, fetal, and placental development and maternal outcomes has been studied in the BEYOND subcohort (Impact of Bariatric surgery on EmbrYONic, fetal and placental) (2021–24, *N* = 91) [10]. The PROMOTE subcohort (2022–25, *N* = 112) was initiated to explore associations between maternal microbiota, immune responses, and one-carbon metabolism in (pre) pregnant women with obesity [11]. Women are enrolled preconceptionally and in the first trimester for microbiota and longitudinal blood sampling.

In blood samples, we investigated biomarkers of the renin–angiotensin–aldosterone system (RAAS) to gain insight in first-trimester cardiovascular adaptation in the Virtual Placenta subcohort (2017–19, *N* = 241). In the ongoing cohort, we collected longitudinal samples to measure maternal cell-free DNA (cfDNA) methylation (2022–24, *N* = 300). Additional biomaterials include maternal plasma and placental tissue postpartum to identify gestational-age and disease-specific methylation patterns. In first-trimester samples (2010–20, *n* = 1916), tryptophan metabolites, homocysteine, and C-reactive protein were analyzed (liquid chromatography-tandem mass spectrometry and immunoassays). Recently, additional maternal hair samples (2022–23, *N* = 160) were collected to assess cortisol and cortisone as biomarkers of maternal stress [12].

### Development of methods for advanced data analysis

In our previous cohort update, we described novel VR-based measurements—embryonic volume (EV), head volume (HV), uteroplacental vascular volume (uPVV), and Carnegie stages, enabling assessment of both growth and morphology [9]. However, manual VR measurements are time-consuming (5–10 minutes), require training, and require specialized equipment, limiting clinical use. Our large collection of first-trimester 3D ultrasounds enables AI-driven extraction of embryonic, fetal, and placental biomarkers. At this stage, both the embryo and placenta fit within a single image, providing a unique opportunity for in-depth analysis of first-trimester developmental processes.

Another recent development is the integration of multimodal data across the periconceptional period, pregnancy, and early postnatal life to enable the development of dynamic digital twins for early diagnosis, prediction, and prevention of cardiovascular and related complications [13].

### Digital lifestyle care interventions

Our previous findings show that determinants of health, such as nutrition, lifestyle, and body mass index (BMI), are associated with reproduction, embryonic and placental development, and pregnancy outcomes, substantiated by the involvement of one-carbon metabolism, cardiovascular and inflammatory pathways, and epigenetics. This supports the need for prevention by (cost)-effective lifestyle interventions. Accordingly, the www.smarterpregnancy.co.uk was developed based on our earlier research (1986–2010) and findings from the Predict study (>2010) [14]. In surveys and randomized controlled trials with (sub)fertile, high-risk, vulnerable, and obese women, we investigated whether digital lifestyle coaching improves parental health and early pregnancy outcomes.

In the next years, Smarter Pregnancy and our other programs will be further developed into an app and toward family-based digital lifestyle coaching. These initiatives promote the self-management of patients and citizens and aim to integrate personalized lifestyle care into medical care, society, and national women’s health strategies.

## Who is in the cohort?

**Figure 2:**
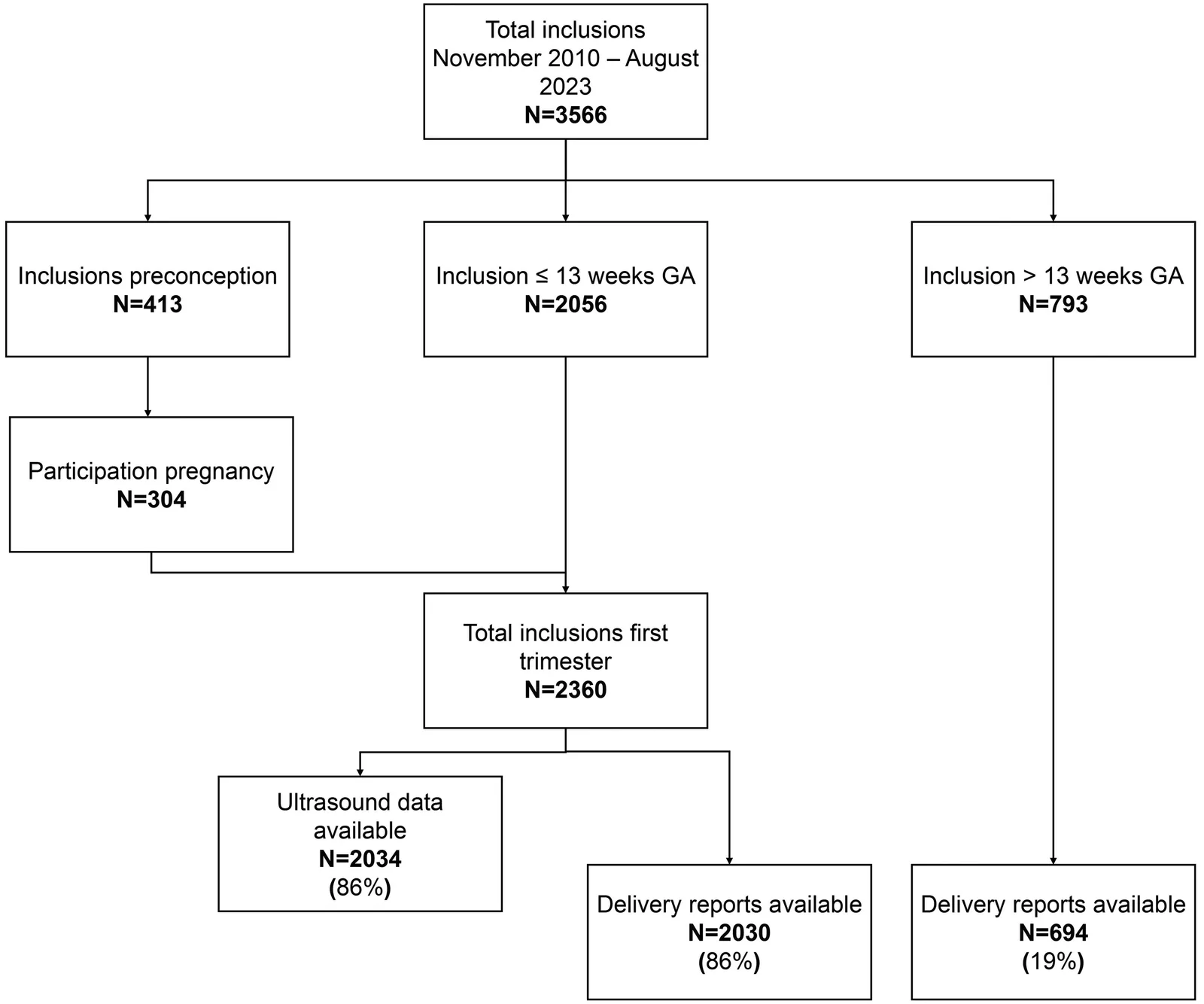
Participant inclusion and exclusion flowchart. Flowchart of included participants from November 2010 until August 2023 (excluding pilot study participants). GA: gestational age.

Between December 2009 and August 2023, 3799 pregnancies were included. The pilot phase (2009–10) included 233 pregnancies from the preconceptional period up to 12 weeks of gestation. From November 2010 to August 2023, 3566 participants were enrolled: 413 preconceptionally, 2056 before 13 weeks, and 793 from 13 weeks to term (Fig. 2). This update includes new results published up to the first quarter of 2025 with data collected through August 2023. Because the cohort is open and recruitment occurs across multiple outpatient clinics, the total number of eligible individuals invited cannot be precisely determined. Table 1 summarizes available questionnaires and samples (excluding the pilot phase).

**Table 1:** Data collection and general characteristics of the participants included between November 2010 and August 2023.

Data collected between November 2010 and August 2023	Women (*N* = 2360)	Men (*N* = 2027)
	*N (%)*	*N (%)*
*First trimester*		
General questionnaires	2101 (89.4)	2009 (99.1)
Food frequency questionnaires	1517 (64.3)	1454 (71.7)
Anthropometric measurements	2252 (95.4)	1500 (74.1)
Venous blood samples	1938 (82.1)	1444 (68.9)
(Serial) ultrasound scans	2034 (86.1)	NA
*24 weeks of gestation*		
Questionnaires	1697 (71.9)	NA
Structural ultrasound records	2014 (85.3)	NA
*36 weeks of gestational to delivery*		
Questionnaires	1501 (63.6)	NA
Delivery reports	2030 (86.0)	NA
Umbilical cord blood	894 (37.9)	NA
Placenta	147 (6.2)	NA
1 year after delivery		
Questionnaires	989 (41.9)	NA
**General characteristics**	**Women**	**Men**
	***Mean (SD) or N (%)***	***Mean (SD) or N (%)***
Age, years	32.3 (4.5)	36.4 (3.5)
Geographical origin		
Dutch	1685 (80.2)	1441 (83.8)
Other western	97 (4.6)	48 (2.8)
Non-western	319 (15.2)	231 (13.4)
Education		
Low	164 (7.9)	252 (14.9)
Middle	753 (36.2)	632 (37.3)
High	1162 (55.9)	812 (47.9)
BMI, kg/m^2^	26.1 (5.1)	26.5 (4.1)
Parity		NA
Nulliparous	1016 (50.0)	
Multiparous	1014 (50.0)	
Mode of conception		NA
Natural	1007 (56.2)	
IVF/ICSI	784 (43.8)	
*First trimester outcome*		NA
Miscarriage	225 (9.8)	
*Pregnancy outcome*		NA
Birthweight, g	3251 (680)	
Gestational age at birth, days	269 (30)	NA
PIH/preeclampsia	181 (7.9)	NA
Preterm birth (<37 weeks GA)	213 (9.3)	NA
Small for gestational age (<p10)	228 (9.9)	NA
Congenital anomaly	72 (3.1)	NA

Update until August 2023, since all participants delivered, and we could include the pregnancy outcome data in this manuscript. SD: standard deviation; IVF: *In vitro* fertilization; ICSI: intracytoplasmic sperm injection; BMI: body mass index; PIH: pregnancy-induced hypertension; GA: gestational age.

## What has been measured?

### Development of new methods for advanced data analysis

We developed the uteroplacental vascular skeleton (uPVS), a computer-assisted biomarker derived by applying a skeletonization algorithm to uPVV data [15]. Each voxel is classified (end-point, vessel-point, bifurcation, or crossing), allowing the calculation of total vascular length. The uPVS revealed impaired vascular development in pregnancies with placenta-related complications, and positive associations with embryonic and fetal growth as well as birth weight percentiles [15].

### Automatic analysis using AI

**Figure 3:**
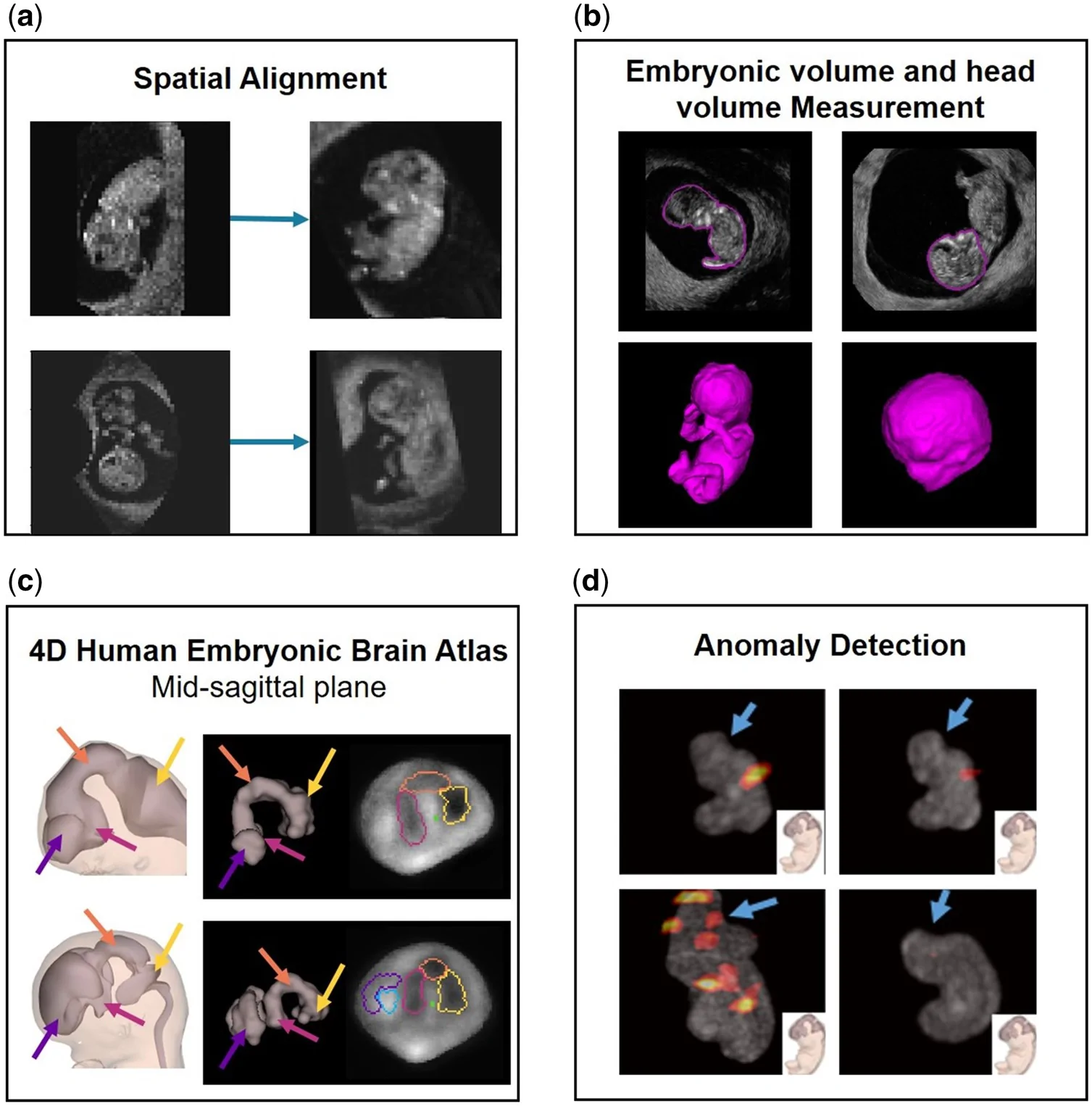
Artificial intelligence–based image analysis. Results of automatic image analysis using artificial intelligence (AI). (a) Extracted mid-sagittal plane after automatic spatial alignment in gestational weeks 10 and 12. (b) Embryonic volume and embryonic head volume measurements, shown as image slices and three-dimensional renderings in gestational week 11. (c) First column: three-dimensional embryo atlas; second and third columns: four-dimensional Human Embryonic Brain Atlas. Purple arrow/outline: lateral ventricles; magenta arrow/outline: third ventricle; orange arrow/outline: cavity of the mesencephalon; yellow arrow/outline: fourth ventricle; green outline: cerebellum; blue outline: choroid plexus. First row: 60 days gestational age; second row: 66 days gestational age. (d) Automated detection of embryonic brain anomalies in gestational week 9, with detected regions highlighted in red.

Table 2 summarizes automated biomarkers developed for first-trimester 3D ultrasonography in the entire cohort. A key challenge is the variable embryonic orientation, which hinders standard plane assessment. To address this, we applied a multi-atlas segmentation and registration approach using AI to standardize orientation and extract the required planes, including the mid-sagittal view (Fig. 3a) [16].

**Table 2:** Overview of artificial intelligence–based methods for early pregnancy ultrasound analysis and their performance in the Predict cohort.

Article	Task	Description method	Dataset size	Gestational age	Results
Bastiaansen *et al*., 2022 [16]	Spatial alignment, detection of mid-sagittal plane	A multi-atlas framework for automatic spatial alignment. During training, the framework learns to align the embryo to a subset of atlases using manually marked crown-rump landmarks. The atlases consist of ultrasound images from multiple subjects across various gestational ages, spatially aligned to a predefined orientation. From this standard orientation, the mid-sagittal plane can be extracted.	Training: 3938 datasets of 874 pregnancies Validation: 1238 datasets of 200 Testing: 1249 datasets of 200 pregnancies	8 + 0–12 + 6	Time per dataset: 16 seconds Correct mid-sagittal: 60% Aligned landmarks: 27% Failed: 13% (due to image quality and acquisition in 89% of the cases)
Bastiaansen *et al*., 2025 [18]	Embryonic volume and embryonic head volume measurements	nnU-net was used, a state-of-the-art segmentation algorithm that is publicly available. After segmentation, the process of determining which voxel is part of the embryo or embryonic head, the volume of segmented region was measured. Additionally, experienced raters scored both VR and AI measurements for correctness.	Training: 648 datasets of 341 subjects (EV), 383 datasets of 261 pregnancies (HV) Testing: 367 datasets of 198 pregnancies (EV), 150 datasets of 123 pregnancies (HV)	EV: 7 + 0–11 + 6 HV: 9 + 0–11 + 6	Time per dataset: 1 minute computation + 1 minute rating Rating EV excellent: AI: 42%, VR: 38 Rating HV excellent: AI: 79%, VR: 17% Intraclass correlation coefficients: EV: 0.99, HV: 0.99
Bastiaansen *et al*., 2026 [19]	4D Human Embryonic Brain Atlas	The atlas was created using a deep learning approach for groupwise template matching and spatiotemporal atlas generation. Our method introduced a time-dependent initial atlas and penalized deviations, preserving age-specific anatomy during rapid development.	Atlas generation: 654 datasets of 308 pregnancies Validation: 97 datasets of 51 pregnancies Testing: 97 datasets of 51 pregnancies	8 + 0–12 + 6	- The resulting atlas matched in terms of anatomy comparable state-of-the-art 3D embryo atlases. - The time-dependent initial atlas and penalized deviations were crucial to produce an anatomically correct atlas.
Zijta *et al*., 2024 [20]	Congenital brain anomaly detection	3D extension of Efficient AD, a state-of-the-art semi-supervised anomaly detection method. Semi-supervised refers to training the method only with normal data, such that it learns to flag anomalous data.	Training: 280 datasets of 280 pregnancies Validation: 59 datasets of 59 pregnancies Testing: 65 datasets of 65 pregnancies Anomalous: 7 datasets of 4 pregnancies	9 + 0–9 + 6	Anomaly detection: AUC: 0.86 Accuracy: 83%

AI: artificial intelligence; AUC: area under the curve; EV: embryonic volume; HV: embryonic head volume; nnU-Net: neural network U-Net; VR: virtual reality.

We also automated volumetric measurements, which were previously performed semi-automatically in VR and required expert training and equipment [17]. Using nnU-Net, a state-of-the-art segmentation method, we measured EV and embryonic HV [18]. Processing time was reduced from 5 to 10 minutes in VR to 2 minutes (1 for computation, 1 for quality control). Expert raters verified AI-generated results, which showed excellent agreement (intraclass correlation coefficient, ICC: 0.996 for EV and 0.997 for HV for future-acquired data; Fig. 3b).

Beyond traditional biomarkers, we developed the four-dimensional (4D) Human Embryonic Brain Atlas, a spatiotemporal model of first-trimester brain development (Fig. 3c). This atlas enhances understanding of normal embryonic growth and its modulation by parental exposures such as maternal BMI. Constructed with AI to capture rapid morphological change, it was validated against existing reference atlases and shown to accurately reflect the gold standard, confirming its reliability [19].

AI also supports early detection of congenital anomalies (e.g. spina bifida, gastroschisis, omphalocele, and anencephaly), providing more time for diagnosis and decision making. Using a semi-supervised approach trained on abundant normal data, we detected congenital brain anomalies with 83% balanced accuracy and an AUC of 0.87 at gestational week 9 [20]. Figure 3d shows examples of regions identified as abnormal.

### Digital lifestyle care interventions

The 26-week lifestyle coaching program (www.smarterpregnancy.co.uk; www.slimmerzwanger.nl) targets diet, folic acid, mental health, exercise, and smoking/alcohol cessation, with weekly assignments, personalized feedback, and goal-setting. Besides its appreciation, improvements were shown for folic acid use, diet, and smoking cessation, which were associated with higher pregnancy chances, larger embryos, and lower maternal blood pressure [21]. These results highlight the potential of app-based tools to support healthier lifestyles in this critical window and substantiate the need for integrating digital lifestyle care into healthcare and society. After demonstrating (cost)effectiveness and recognition by the National Institute for Public Health and the Environment (RIVM) in 2020 and 2025, Smarter Pregnancy is being integrated into standard and digital patient care.

## What has been found? Key findings and publications

Since the previous update, new findings have emerged from the expansion of biomarker research, AI-based imaging analyses, and implementation of digital lifestyle care. Key findings are summarized below and Tables S1 and S2.

### Determinants of maternal and paternal periconceptional health and embryonic development

Maternal obesity accelerates pre-implantation embryo development but slows embryonic growth, while underweight has the opposite effect, especially in female embryos [22]. Maternal multi-micronutrient supplement use, including folic acid, increases embryonic growth and lowers the risk of small-for-gestational-age (SGA). Periconceptional maternal and paternal alcohol use was associated with smaller fetal size at 20 weeks, while smoking delayed development and reduced embryonic and fetal growth throughout pregnancy [23]. These findings underscore the importance of addressing periconceptional lifestyle factors.

### Molecular mechanisms, epigenetics, cardiovascular pathways

Analysis of RAAS biomarkers in first-trimester samples demonstrated that higher RAAS activity was associated with reduced late fetal growth, lower birthweight, and an increased risk of SGA neonates [24]. Altered maternal tryptophan (kynurenine) metabolism in the first trimester, characterized by lower tryptophan and higher kynurenine and quinolinic acid, was associated with impaired placental vascular development, reduced embryonic and fetal growth, and an increased risk of SGA neonates [25]. Meanwhile, the absence of a corpus luteum decreases RAAS activity and placental vascular development and is associated with preeclampsia and diabetes. Uteroplacental vascular morphology, quantified using the newly developed uPVS, revealed impaired vascular development in pregnancies with placenta-related complications and positive associations with embryonic and fetal growth and birthweight [15, 26]. In addition, recent analyses of maternal cfDNA methylation showed that placental-derived methylation patterns are influenced by maternal nutritional and metabolic factors, highlighting the role of one-carbon metabolism in early epigenetic programming [27]. Importantly, cfDNA reflects a mixture of placental-derived fragments and maternal immune cell DNA, providing a minimally invasive window into both placental and maternal biological process [27]. In addition, maternal stress, measured by elevated hair cortisol concentrations, was associated with reduced embryonic growth [28]. These findings build on earlier observations by identifying specific metabolic and endocrine pathways connecting maternal exposures to embryonic development.

### Reproductive performance and pregnancy outcomes

In recent analyses, we demonstrated that a larger oocyte area was associated with higher-quality and faster-developing embryos, while a greater blastocyst surface increased the chance of ongoing pregnancy [29, 30]. Fresh embryo transfer was associated with lower relative birth weight compared to natural conception. However, first-trimester uteroplacental development and embryonic growth did not differ between frozen-thawed and fresh transfers. Post-implantation growth varied by sex, and pre-implantation kinetics differed across culture media. Higher maternal and paternal BMI accelerated pre-implantation development without affecting embryo quality, whereas low maternal vegetable intake and paternal smoking reduced implantation potential after IVF/ICSI [31].

### Digital lifestyle care interventions

Participants who used Smarter Pregnancy showed significant improvements in key behaviors during the preconception and early pregnancy. Participants reported higher fruit and vegetable intake, improved diet quality, increased folic acid use, and stronger adherence to lifestyle recommendations, particularly when partners also participated and in women with overweight or obesity. Participation was further associated with lower maternal blood pressure during pregnancy, underscoring the potential of digital coaching to address modifiable risks and improve maternal health and pregnancy outcomes [21].

## What are the main strengths and weaknesses?

The main strength is the unique prospective periconceptional design, embedded in tertiary care. Couples complete questionnaires, provide blood samples, and women undergo longitudinal 2D/3D ultrasounds from 6 weeks onward using high-frequency probes and VR. With >3566 couples, this is the first cohort to validate AI methods for first-trimester image analysis. Integrated imaging, biochemical, dietary, molecular, and (epi)genetic data provide insights into reproductive outcomes, growth, and birth. Importantly, findings have been translated into clinical innovation through the Smarter Pregnancy program. Although the tertiary care setting ensures high internal validity, it may limit external generalizability. External validation, including Generation R Next, and further intervention studies will help confirm and extend our findings.

## Can I get hold of the data? Where can I find out more?

Colleagues interested in collaboration can submit a brief research proposal. Approval is based on quality and feasibility, assessed by the Predict Management team. A formal contract outlining mutual obligations is required. For inquiries, contact the principal investigator (r.steegers@erasmusmc.nl) or predictstudie@erasmusmc.nl. All publicly available code for the developed AI models, including the 4D Human Embryonic Brain Atlas, can be found online [https://gitlab.com/radiology/prenatal-image-analysis].

## Ethics approval

The Predict study (MEC-2004–227) and all subcohort studies were separately approved by the Central Committee on Research in The Hague and the local Medical Ethics Committee of the Erasmus Medical Center (MC) in Rotterdam, The Netherlands.

## Supplementary Material

dyag156_Supplementary_Data
