# Supplementary material for: Cohort Profile Update: the Rotterdam Periconceptional Cohort—biomarkers, lifestyle intervention, and artificial intelligence
Source: Int J Epidemiol. 2026 Aug 19;55(5):dyag156. doi: 10.1093/ije/dyag156 (PMC13487898; doi:10.1093/ije/dyag156)
Supplement: Supplementary file 1 [file dyag156_supplementary_data.docx]

**Supplementary Table S1: Part 1): Maternal and paternal health factors**

**and significant associations with reproductive (preconceptional), pregnancy and neonatal outcomes**

a) *Reproductive (preconceptional) outcomes*

|  | **Follicle-Oocyte** | | | | **Sperm** | | **Pre-implantation Embryo** |
| --- | --- | --- | --- | --- | --- | --- | --- |
|  | **Oolemma area (µm^2^) at pronuclear appearance (tPNa)**  **Beta (95% CI) or beta; SE** | **Rate of area shrinking**  **(µm2/hour)**  **Beta (95% CI) or beta; SE** | **Follicles/Oocytes** | **Odds of a successful fertilization**  **OR (95% CI)** | **TMSC, total motile sperm**  **Beta (95% CI) or beta; SE** | **Morphokinetic parameters(h):** **Beta (95% CI) or beta; SE** | **Pre-implantation Embryo Quality measured by a time-lapse morphokinetic selection algorithm (KIDScore)**  **Beta (95% CI), beta; SE, OR [95% CI]** |
| **Maternal health factors** |  |  |  |  |  |  |  |
| **Plasma prorenin concentrations (pg/ml) ^30^** | -1.37 (-2.48, -0.26) |  | **Pre-transfer**  No. of follicles (n)  3.75 (2.89, 4.59)  Total follicle diameter (mm)  48.80 (35.49, 62.10)  No. of oocytes (n)  3.83 (2.90, 4.75)  Metaphase ll oocytes (n)  2.09 (1.43, 2.75)  Total fertilized oocytes (n)  0.98 (0.54, 1.41)  **Post-transfer**  Positive β-hCG-test  1.79 [1.06, 3.08] |  |  |  |  |
| **Paternal health factors** |  |  |  |  |  |  |  |
| **BMI^8^** |  |  |  |  | count:  −2.48; 1.53  Fertilization rate:  −0.01 ;0.004 | −0.19 (−0.33, −0.04) |  |
| **Smoking^31^** |  |  |  |  |  |  | 0.53 [0.33, 0.86] |

**Abbreviations**: 95% CI – 95% confidence interval; β-hCG – beta human chorionic gonadotropin; Beta (β) – regression coefficient; BMI – body mass index; h – hours; KIDScore – known implantation data score; mm – millimeters; n – number; OR – odds ratio; pg/mL – picograms per milliliter; SE – standard error; TMSC – total motile sperm count; tPNa – time of pronuclear appearance; µm² – square micrometers.

b) *Pregnancy outcomes: Embryo and fetus*

|  | **Embryo** | | |  | **Fetus** | | | | |
| --- | --- | --- | --- | --- | --- | --- | --- | --- | --- |
|  | **CRL**  **Beta (95% CI) or beta; SE** | **EV**  **Beta (95% CI) or beta; SE** | **Carnegie stage**  **Beta (95% CI) beta; SE; OR [95% CI]** | **HC**  **Beta (95% CI) or beta; SE** | **AC**  **Beta (95% CI) or beta; SE** | **FL**  **Beta (95% CI) or beta; SE** | **EFW**  **Beta (95% CI) or beta; SE** | **BPD**  **Beta (95% CI) or beta; SE** | **HV/EV**  **Beta (95% CI) or beta; SE** |
| **Maternal health factors** |  | | |  |  |  |  |  | |
| **Underweight (BMI < 18.5)^22^**  Embryo (female) |  | 0.13 (0.03, 0.23) | 0.79 (0.25, 1.31)  1.39 (0.74, 2.05) |  |  |  |  |  |  |
| **Obesity (BMI ≥ 30) and embryo (female)^22^** |  | -0.05 (-0.09, -0.01) |  |  |  |  |  |  |  |
| **Smoking ≥ 10 cigarettes/day^32^**  Whole population  after IVF/ICSI treatment |  |  | -0.352 (-0.648, -0.057)  -0.510 (-0.834, -0.186) |  |  |  |  |  |  |
| **Smoking ^32^** |  |  |  | 0.290 (0.065, 0.514) |  | -0.321 (-0.641, 0.001) |  |  |  |
| **Moderate alcohol usage (>3-≤30 gs)^23^** | 0.09; 0.05 |  |  | -1.85; 0.84 | -2.65; 0.93 | -0.56; 0.22 | -9.36; 4.35 | -1.85; 0.84 |  |
| **Bariatric bypass surgery^33^** |  |  |  | 0.982 (0.972, 0.992) | 0.963 (0.953, 0.974) | 0.964 (0.951, 0.977) | 0.954 (0.927, 0.973) | 0.979 (0.966, 0.992) |  |
| **Multiple-micronutrient supplement use^34^** | 0.052 (0.012, 0.090) | 0.022 (0.002, 0.042) |  |  |  |  |  |  |  |
| **Intake of fatty acids^35^**   i. eicosapentaeonic acid (EPA) ii. docosahexaenoic acid (DHA), iii. Other polyunsaturated fatty acids (PUFAs) iv. Non PUFA-rich diet v. DHA and EPA-rich diet vi. PUFA-rich diet |  |  |  |  |  |  |  |  | i. -0.621 (-1.190, -0.051) ii. -0.431 (-0.819, -0.044) iii. -0.106 (-0.170, -0.043) iv. 1.031e − 02(2.966e − 03,1.764e − 02) v. -4.917e − 03(-9.938e − 03,1.027e − 04) vi. 6.783e − 03(-1.098e − 02, -2.582e − 03) |
| **Red blood cell Folate(nmol/l)^35^** |  |  |  | 2.745 (0.340, 5.149) |  |  |  |  | 0.866(0.286, 1.447) |
| **Plasma 5-hydroxytryptophan-HTP(nmol/l)^25^** | -0.015 (-0.028, -0.001) | -0.009 (-0.016, -0.003) |  |  |  |  |  |  |  |
| **Plasma 5-hydroxytryptophan/tryptophan ratio^25^** | -0.843 (-1.597, -0.088) | -0.529 (-0.902, -0.157) |  |  |  |  |  |  |  |
| **Plasma Prorenin concentration (pg/ml)^24^** |  |  |  |  |  |  | -0.07 (-0.11, -0.03) |  |  |
| **Paternal health factors** |  |  |  |  |  |  |  |  |  |
| **Alcohol use^23^** |  |  |  |  | 0.033; 0.01 |  | 0.131; 0.06 |  |  |

**Abbreviations**: 95% CI – 95% confidence interval; AC – abdominal circumference; Beta (β) – regression coefficient; BMI – body mass index; BPD – biparietal diameter; CRL – crown–rump length; DHA – docosahexaenoic acid; EPA – eicosapentaenoic acid; EFW – estimated fetal weight; ET – embryo transfer; EV – embryonic volume; FL – femur length; HC – head circumference; HV/EV – head volume–to–embryonic volume ratio; ICSI – intracytoplasmic sperm injection; IVF – in vitro fertilization; MTD – mesencephalon total diameter; nmol/L – nanomoles per liter; OR – odds ratio; pg/mL – picograms per milliliter; PUFA – polyunsaturated fatty acid; SE – standard error; tHTP – 5-hydroxytryptophan; TTR – telencephalon thickness right; TTL – telencephalon thickness left; µmol/L – micromoles per liter; 3√ml – cube root milliliters.

c) *Pregnancy outcomes: Placenta and uterine environment*

|  |  |  | **Placenta – first trimester** |  | **Placenta – at delivery** | **Uterine environment** |
| --- | --- | --- | --- | --- | --- | --- |
|  | **PV**  **Beta (95% CI) or beta; SE** | **uPVV**  **Beta (95% CI) or beta; SE** | **uPVS**  **Beta (95% CI) or beta; SE** | **uPVV/PV**  **Beta (95% CI) or beta; SE** | **Weight of the delivered placenta; Effect size (95% CI)** | **UtA, uterine artery; RI, resistance index** |
| **Maternal factors** |  |  |  |  |  |  |
| **Placental growth factor (PlGF)^36^, serum** | 0.53 (0.34, 0.73) | 0.39 (0.15, 0.64) | Bifurcation points: 4.64 (0.04, 9.25)  Crossing points: 4.01 (0.65, 7,37)  Total length: 13.33 (3.09, 23.58)  Thickness: 0.05 (0.01, 0.08)  Density endpoints: -1.73 (-3. 19, -0.28)  Density bifurcation points: -0.76 (-1.38, -0.14) |  |  |  |
| **soluble fms-like tyrosine kinase-1/PlGF ratio^36^** | -0.35 (-0.53, -0.16) |  |  |  |  |  |
| **Soluble endoglin/PlGF ratio^36^** | -0.26 (-0.44, -0.08) | -0.30 (-0.52, -0.09) | Bifurcation points: -4.08 (-7.87, -0.30)  Crossing points: -2.80 (-5.59, -0.01)  Vessel points: -9.20 (-17.89, -0.53)  Total length: -10.03 (-18.50, -1.56)  Thickness: -0.03 (-0.06, -0.01)  Density endpoints: 1.38 (0.18, 2.58)  Density bifurcation points: 0.54 (0.03, 1.06) |  |  |  |
| **Imaging markers representing absolute placental volume development and absolute utero-placental vascular volume development^15^**  RI ∛ uPVV (cm^3^) |  |  |  |  | 151.30 (52.31, 250.28) |  |
| **Imaging markers representing absolute morphologic development of the utero-placental vasculature^15^**  RI √uPVS total length (mm) |  |  |  |  | 3.23 (0.28, 6.17) |  |
| **Imaging markers representing density of vascular branching^15^**  RI log density of end points (n/cm^3^) |  |  |  |  | -93.71 (-169.05, -18.37) |  |
| **First-trimester hemodynamic adaptation (In placenta-related complications)^6^**   i. Increase in the UtA PI trajectory ii. Increase in the UtA RI trajectory | i. 0.559 (−0.841, −0.278) ii. −0.579 (−0.878, -0.280) | i. - ii. −0.301 (-0.578, −0.023) |  |  | i. −0.502 (−0.922, −0.082) ii. −0.435 (−0.839, −0.032) |  |

**Abbreviations:** 95% CI – 95% confidence interval; PI – pulsatility index; PlGF – placental growth factor; PV – placental volume; RI – resistance index; SE – standard error; tHcy – total homocysteine; uPVS – utero-placental vascular skeleton; uPVV – utero-placental vascular volume; uPVV/PV – utero-placental vascular volume to placental volume ratio; UtA – uterine artery; UtA-PI – uterine artery pulsatility index; UtA-RI – uterine artery resistance index; UmbA-PI – umbilical artery pulsatility index; µmol/L – micromoles per liter; pg/mL – picograms per milliliter.

d) *Neonatal outcomes*

|  | **Birthweight**  **Beta (95% CI) or beta; SE** | **SGA**  **OR [95% CI]** | **Prematurity**  **OR [95% CI]** | **Adverse outcome**  **OR [95% CI]** |
| --- | --- | --- | --- | --- |
| **Maternal health factors** |  |  |  |  |
| **Gastric bypass surgery^33^** | -241.4 (-342.7, -140.0) (g)  -17.9 (-25.1, -10.6) (hoftizer percentiles) |  |  |  |
| **5-hydroxytryptophan/tryptophan ratio^25^** |  | 1.006 (1.000, 1.013) |  |  |
| **Plasma Prorenin concentration (pg/ml)^24^** | -0.28, (-0.55, -0.01) | Log transformed prorenin levels  10.47 [2.49, 53.21] |  |  |
| **>1 corpus luteum^24^** | -0.17 (-0.32, -0.01) |  |  |  |
| **Total follicle diameter^24^** | -0.61 (-1.01, -0.20) |  |  |  |
| **First-trimester hemodynamic adaptation (pregnancies without placenta-related complications)^6^**  Higher mean arterial blood pressure | 0.398 (0.049, 0.748) |  |  |  |

**Abbreviations:** 95% CI – 95% confidence interval; CRL – crown–rump length; EV – embryonic volume; g – grams; µmol/L – micromoles per liter; OR – odds ratio; pg/mL – picograms per milliliter; SE – standard error; SGA – small for gestational age.

**Supplementary Table S2: Part 2: Significant results of the exploration of molecular mechanisms, epigenetics, cardiovascular pathways**

|  | **Associations** | **Beta (95% CI), beta; SE, OR [95% CI]** |
| --- | --- | --- |
| **Maternal health factors and biomarkers** |  |  |
| **Vulnerability risk score^28^** | i. Hair Cortisol (pg/mg) ii. Hair Cortisone (pg/mg) iii. Plasma concentrations Tryptophan (µmol/L) | i. 0.366 (0.010, 0.722) ii. 0.897 (0.102, 1.691) iii. -1.637 (-2.693, -0.582) |
| **Intake of ultra-processed foods^37^**   i. All ii. Normal BMI | Plasma homocysteine levels (umol/L) | i. 1.31 (0.38, 2.23) ii. 1.07 (0.06, 2.07) |
| **Energy intake (kJ/day)^38^** | Plasma concentrations of:   i. Tryptophan (µmol/L) ii. 5-Hydroxytryptophan (nmol/L) | i. -0.00 (-0.00, -0.00) ii. -0.00 (-0.00, -0.00) |
| **Hair cortisol (pg/mg)^28^** | Embryo: EV | -0.010 (-0.017, -0.002) |
| **Protein intake (g/day)^38^** | Plasma concentrations of:   i. Tryptophan (µmol/L) ii. 5-Hydroxytryptophan (nmol/L) | i. 0.12 (0.07, 0.17) ii. 0.01 (0.00, 0.02) |
| **Plasma homocysteine (µmol/l)^38^** | Plasma concentrations of:   i. Kynuerine (µmol/L) ii. 5-Hydroxytryptophan (nmol/L) | i. (0.00, 0.02) ii. 0.07 (0.03, 0.11) |
| **Utero-placental vasculature (1^st^ trimester)^26^**   i. √uPVS endpoints ii. √PVS bifurcation points iii. √uPVS crossing points. iv. √uPVS vessel points v. √Total length vi. √Average thickness | Embryo: CRL | i. 0.017 (0.009, 0.025) ii. 0.012 (0.006, 0.018) iii. 0.017 (0.008, 0.025) iv. 0.005 (0.002, 0.008) v. 0.007 (0.004, 0.010)   - |
| **Utero-placental vasculature (1^st^ trimester)^26^**   i. √uPVS endpoints ii. √PVS bifurcation points iii. √uPVS crossing points. iv. √uPVS vessel points v. √Total length vi. √Average thickness | Embryo: EV | i. - ii. 0.008 (0.004, 0.012) iii. 0.005 (0.004, 0.008) iv. 0.008 (0.004, 0.012) v. 0.002 (0.001, 0.004) vi. 0.003 (0.002, 0.005) |
| **Utero-placental vasculature (1^st^ trimester)^26^**   i. √uPVS endpoints ii. √PVS bifurcation points iii. √uPVS crossing points. iv. √uPVS vessel points v. √Total length vi. √Average thickness | Embryo: Carnegie stage | i. 1.233 [1.013, 1.502] ii. 1.139 [1.002, 1.295] iii. 1.196 [1.001, 1.429] iv. 1.062 [1.001, 1.126] v. 1.077 [1.005, 1.154]   - |
| **Utero-placental vasculature (1^st^ trimester)^26^**   i. √uPVS endpoints ii. √PVS bifurcation points iii. √uPVS crossing points. iv. √uPVS vessel points v. √Total length vi. √Average thickness | Fetus: EFW | i. 3.379 (0.945, 5.813) ii. 2.134 (0.370,3.897) iii. 3.207 (0.744, 5.670) iv. 0.856 (0.094, 1.618) v. 1.205 (0.317, 2.092) vi. - |
| **In vitro environmental factors** |  |  |
| **Oolemma surface area and human pre-implantation embryo development and usage**  (in ICSI) Oolemma area at t0 (µm^2^/hour) | i. Oolemma, rate of area shrinking (µm2/hour) ii. Odds of successful fertilization | i. -12.55 (-14.55, -10.55) ii. 0.57 [0.48, 0.67] |
| **Oocyte area and fertilization rate^29^**  (In ICSI) Oocyte area ­(10^−3^ µm^2^) | Odds of successful fertilization | adjusted for  used/discarded  1.576 [1.274, 1.951] |

**Abbreviations:** BMI (body mass index); PV (placental volume); uPVV (utero-placental vascular volume); PV/uPVV (placental volume-to-vascular volume ratio); tPNa (time of pronuclear appearance); tPNf (time of pronuclear fading); t2 (first cell cleavage time); CI (confidence interval); SE (standard error); OR (odds ratio); µmol/L (micromoles per liter); nmol/L (nanomoles per liter); pg/mg (picograms per milligram); R (Pearson correlation coefficient); TESE (testicular sperm extraction); ET (embryo transfer); PI (pulsatility index); UmbA (umbilical artery); IVF (in vitro fertilization); ICSI (intracytoplasmic sperm injection);
